# Expansion of Host Regulatory T Cells by Secreted Products of the Tapeworm *Echinococcus multilocularis*

**DOI:** 10.3389/fimmu.2020.00798

**Published:** 2020-05-08

**Authors:** Justin Komguep Nono, Manfred B. Lutz, Klaus Brehm

**Affiliations:** ^1^Institute of Hygiene and Microbiology, University of Würzburg, Würzburg, Germany; ^2^Division of Immunology, Health Science Faculty, University of Cape Town, Cape Town, South Africa; ^3^The Medical Research Centre, Institute of Medical Research and Medicinal Plant Studies, Ministry of Scientific Research and Innovation, Yaounde, Cameroon; ^4^Institute of Virology and Immunobiology, University of Würzburg, Würzburg, Germany

**Keywords:** helminth, cestode, *Echinococcus*, regulatory T cells, activin, immunomodulation, excretory/secretory, IL-10

## Abstract

**Background:**

Alveolar echinococcosis (AE), caused by the metacestode larval stage of the fox-tapeworm *Echinococcus multilocularis*, is a chronic zoonosis associated with significant modulation of the host immune response. A role of regulatory T-cells (Treg) in generating an immunosuppressive environment around the metacestode during chronic disease has been reported, but the molecular mechanisms of Treg induction by *E. multilocularis*, particularly parasite immunoregulatory factors involved, remain elusive so far.

**Methodology/Principal Findings:**

We herein demonstrate that excretory/secretory (E/S) products of the *E. multilocularis* metacestode promote the formation of Foxp3^+^ Treg from CD4^+^ T-cells *in vitro* in a TGF-β-dependent manner, given that this effect was abrogated by treatment with antibody to mammalian TGF-β. We also show that host T-cells secrete elevated levels of the immunosuppressive cytokine IL-10 in response to metacestode E/S products. Within the E/S fraction of the metacestode we identified an *E. multilocularis* activin A homolog (EmACT) that displays significant similarities to mammalian Transforming Growth Factor-β (TGF-β/activin subfamily members. EmACT obtained from heterologous expression failed to directly induce Treg expansion from naïve T cells but required addition of recombinant host TGF-β to promote CD4^+^ Foxp3^+^ Treg conversion *in vitro*. Furthermore, like in the case of metacestode E/S products, EmACT-treated CD4^+^ T-cells secreted higher levels of IL-10. These observations suggest a contribution of EmACT to *in vitro* expansion of Foxp3^+^ Treg by the *E. multilocularis* metacestode. Using infection experiments we show that intraperitoneally injected metacestode tissue expands host Foxp3^+^ Treg, confirming the expansion of this cell type *in vivo* during parasite establishment.

**Conclusion/Significance:**

In conclusion, we herein demonstrate that *E. multilocularis* larvae secrete factors that induce the secretion of IL-10 by T-cells and contribute to the expansion of TGF-b-driven Foxp3^+^ Treg, a cell type that has been reported crucial for generating a tolerogenic environment to support parasite establishment and proliferation. Among the E/S factors of the parasite we identified a factor with structural and functional homologies to mammalian activin A which might play an important role in these activities.

## Introduction

The metacestode larval stage of the fox-tapeworm *Echinococcus multilocularis* is the causative agent of alveolar echinococcosis (AE), one of the most dangerous zoonoses world-wide (1, 2). Intermediate hosts (rodents and, occasionally, humans) usually get infected by oral ingestion of infectious eggs that contain the oncosphere larva. Upon hatching in the small intestine and penetration of the intestinal wall, the oncosphere gains access to the host organs and, almost exclusively within the liver, develops into the cyst-like metacestode, following a process of stem cell-driven metamorphosis (3, 4). The multi-vesicular *E. multilocularis* metacestode tissue subsequently grows infiltratively, like a malignant tumor, into the surrounding host tissue, eventually leading to organ failure and host death (2). In later stages of the disease, metastases can occur in secondary organs, which is probably due to the distribution of parasite stem cells via bloodstream and the lymphatic system (3). yst-like rniztablished in Kosizng the internship.ace during her stay.in cooperation with very talented young investigator nataIn mice, the initial establishment phase of the parasite (the oncosphere-metacestode transition) is typically accompanied by a potentially parasitocidal, Th1- dominated immune response which, in permissive hosts, is skewed toward a permissive Th2-dominated immune response during the chronic phase of the disease (5). Current treatment options against AE are very limited and include surgery, which can only be applied in few cases, and/or chemotherapy with benzimidazoles (2). However, due to significant adverse side effects, only parasitostatic doses of these compounds can be applied and, consequently, the drugs often have to be administered lifelong (2). These limitations in current AE therapy underscore an urgent need for the development of novel anti-parasitic measures.

During asexual multiplication, the *E. multilocularis* metacestode tissue persists for prolonged periods of time in close contact to immune effector cells without being expelled by the host immune response (5). Immunosuppressive mechanisms, provoked by parasite surface structures and/or excretory/secretory (E/S) products, have thus been proposed to support long-term persistence of the parasite within the host (5–8). Accordingly, PBMCs of patients with active AE and host cells in the vicinity of parasite liver lesions in mice produce elevated levels of the immunosuppressive cytokines TGF-β and IL-10. These cytokines are believed to play important roles in the pathophysiology of AE (9–11). Furthermore, immune effector cells from *E. multilocularis*-infected hosts typically display impaired immune reactivity (9, 12–16) whereas those from hosts with degenerating parasite tend to recover immune responsiveness (17). Moreover, host immune-stimulation during an infection can lead to considerably reduced disease progression (18, 19). Although the molecular and immunological basis for the immune suppression in AE is largely elusive so far, parasitic helminths as a whole have repeatedly been reported to exploit the host immune system’s own self-regulatory signaling pathways for successful establishment of an infection and long-term persistence within the host (20).

Of particular importance for regulation in mammalian immune responses are signals delivered by TGF-β superfamily members. On the basis of sequence similarities, two cytokine sub-families can be distinguished within this superfamily: the TGF-β/activin sub-family and the bone morphogenetic protein (BMP) sub-family (20). The former subfamily has gathered considerable interest concerning mechanisms of immune homeostasis maintenance (21, 22). Produced as large pro-forms consisting of an N-terminal signal peptide, followed by a pro-peptide separated by a furin recognition motif from the C-terminal active peptide (∼15 kDa), TGF-β/activin ligands are secreted as dimers of their active peptide, following cleavage of the signal sequence and pro-peptides (23). Two particularly relevant proteins within the TGF-β/activin subfamily, TGF-β1 and activin A (i.e., inhibin beta A homodimers), have drawn considerable attention in the search for mechanisms that lead to an impairment of immune effector cell functions and, ultimately, to an expansion of tolerogenic cells (21, 22). Both cytokines were reported to impair the function of dendritic cells (DC), NK cells, macrophages, and T-cells, and stimulate the expansion of regulatory DC and T-cells (21, 22).

During echinococcosis, the impaired host immune response is paralleled by an increased expression of TGF-β signaling components in periparasitic host cells and tissues (10, 11, 16, 24–27) with the expansion of tolerogenic CD4^+^ CD25^+^ Foxp3^+^ Treg cells (6, 15, 28–32). *Echinococcus* antigens can stimulate the expression of CD25 by CD4^+^ T helper cells from AE infected patients, contributing to the differentiation into Treg (33). Using a murine system of intraperitoneal AE (secondary echinococcosis), Mejri et al. (15) reported increased percentages of CD4^+^CD25^+^ T-cells in the peritoneum of *E. multilocularis* infected mice at an advanced (chronic) stage of the disease, when compared to non-infected mice. This group also found Foxp3 gene expression to be elevated in these cells and a higher frequency of CD4^+^CD25^+^Foxp3^+^ Treg cells in the peritoneum and the spleen of *E. multilocularis*-infected mice (15, 28). Seminal studies from Gottstein’s group (28–30) then convincingly revealed Foxp3 + Treg as key players in the immunoregulatory processes that facilitate the establishment and persistence of the *E. multilocularis* metacestode in mammalian hosts. Consistent with such observations is our previous report of the ability of *E. multilocularis* metacestode E/S products (MVE/S) to expand host Treg *in vitro* (6). This ultimately suggests that Treg expansion during AE, as increasingly reported in the literature, could go beyond a simple homeostatic balancing mechanism.

In the present study, we specifically followed up on these observations to further investigate the ability of the *E. multilocularis* metacestode to increase host Tregs. We report on parasite E/S factors which include a TGF-β superfamily ligand, EmACT (*E. multilocularis* Activin), that are released by the metacestode and promote the ability of host TGF-β to induce Treg conversion and the production of IL-10 by host T-cells. Our data support a role of parasite-derived factors in the impairment of host immune response during AE and identify a parasite activing-homolog as a potential driver of host immune suppression by the *E. multilocularis* metacestode.

## Materials and Methods

### Animals and Ethics Statement

Wild type C57Bl/6 mice and Mongolian jirds were purchased from Charles River and housed at the local animal facilities of the Institute of Hygiene and Microbiology and the Institute for Virology and Immunobiology of the University of Würzburg (Germany) at least 1–2 weeks before experimentation. OT-II mice (TCR transgenic mice where CD4^+^ T cells are specific for I-A^*b*^ presentation of OVA_323–339_ peptide) were kindly provided by Francis Carbone, Melbourne, Australia. OT-II mice were crossed with Rag-1^–/–^ mice on a C57Bl/6 background, a generous gift from Thomas Winkler, Erlangen, Germany. Animal handling and experimentation was compliant to the European and German regulations on the protection of animals (*Tierschutzgesetz*). Local ethics committee of the government of Lower Franconia provided ethical approval of the study (Regierung von Unterfranken, 55.2-2531.01-31/10 and 55.2-2531.01-26/13).

### *In vitro* Maintenance of *E. multilocularis* Metacestode and Collection of E/S Products

*Echinococcus multilocularis* metacestodes were isolated, separated from host contaminants and axenically cultivated as previously described (6). For the collection of E/S products, metacestode vesicles were kept under axenic conditions for 10 days, washed three times in 1 x PBS and resuspended in DMEM10redox i.e., DMEM ^+^ GlutamaxTM, GIBCO supplemented with 10% Fetal Bovine Serum Superior (Biochrom AG), 100 μg/ml penicillin/streptomycin (PenStrep solution, Biochrom AG), 20 μg/ml Levofloxacin (Tavanic, Sanofi-Aventis), β-mercapthoethanol (143 μM, Sigma-Aldrich, cat. M6250), 10 μM Bathocuproine disulfonic acid (Sigma, cat. B-1125) and 100 μM L-Cysteine (Sigma, cat. C-1276) under axenic conditions [see (6) for description of conditions]. After 48 h of culture, the supernatants containing the MVE/S were collected and filtered through a 0.2 μm sieve (Filtropur S filter, SARSTEDT). The total amount of E/S product proteins was determined as previously defined (6) and the E/S products stored at −80°C until use.

### Injection of *E. multilocularis* Metacestodes and *in vivo* Follow-Up

#### Preparation of Parasite Material and Injections

For *in vivo* assays, metacestode vesicles were obtained from 5 unrelated and previously infected Mongolian jirds (*Meriones unguiculatus*). The absence of contamination with host cells was ensured and confirmed and 500 ul of acephalic cysts of PBS solution (as mock were injected to mice as previously defined (6).

#### Peritoneal Lavage and Cell Collection

Peritonea were flushed from animals sacrificed at different time points post injection (i.e., 3, 7, 14, and 42 days) to recover peritoneal exudate cells and animals were dissected to fully retrieve parasite material. Peritoneal exudates were pooled by groups of 3 for naïve mice and used from single animals for infected mice. After filtration through a 70 μm cell strainer (BD Biosciences), red blood cells were lysed by incubation of the suspension in ammonium chloride and the obtained pellet was resuspended and counted for cell numbers.

#### Flow Cytometry

Staining was performed on peritoneal exudate cells using the following anti-mouse antibodies and flurorochrome conjugates: anti-CD4 with Biotin (Miltenyi Biotec), anti-CD25 with PE (eBioscience) and anti-Foxp3 with APC (Miltenyi Biotec). Secondary staining for biotinylated CD4 was done with either FITC- or Pe-Cy5-conjugated streptavidin (BD Biosciences). As isotype control of activated/regulatory T-cells, mouse IgG1 K isotype (APC, Miltenyi Biotec) and Rat IgG1 K Isotype (PE, eBioscience) were used. The staining procedure was executed as previously described (6) and results acquired on a cytometer (FACSCalibur^TM^, Beckton Dickinson) and analyzed on FlowJo software (Tree Star, United States).

### *In vitro* Treg Suppression Assay

The ability of proliferating naïve T cells to be suppressed by isolated CD25 + regulatory T cells was assessed as previously defined (thesis). Briefly, healthy spleens were recovered and peritoneal exudated cells of parasite infected mice (7 days after infection) were retrieved (pooled for 20 infected mice). CD4^+^ cells were isolated by negative selection (EasySep Enrichment Kit, Stem Cell Technologies) to a purity of >90% following the manual instructions. Isolated CD4^+^ T-cells were labeled with CD4 antibody (Biotin, Miltenyi Biotec) and CD25 antibody (PE, eBioscience) and Pe-Cy5-conjugated streptavidin (BD Biosciences) used for secondary staining of CD4-Biotin. CD25^–^ and CD25^+^ cells from the CD4 + T cell suspensions were sorted on a MoFlo high-speed sorter (Cytomation). Sorted CD25^–^ splenic T cells (responders) were incubated with a CFSE-containing solution (2 μM, CFDA SE, Molecular Probes/Invitrogen) at 37°C for 10 min. After incubation, the cell suspension was washed in culture medium (R10) before subsequent use.

Next, antigen presenting cells (APC) were isolated from spleen of naïve mice as previously described (thesis). APC were irradiated with 20 Grays (Faxitron, CellRad) and seeded (2 × 10^5^) in co-culture with responder T cells in the presence of pre-coated anti-CD3 (1 ug/ml, eBioscience). irradiated APCs was then cultured in CD3 antibody (1 ug/ml, eBioscience) pre-coated 96-well round-bottom plates with responders (2 × 10^4^ CFSE-labeled splenic CD4^+^CD25^–^ cells) and CD4^+^CD25^+^ regulatory T cells for 5 days. After incubation, the cells were harvested, washed, acquired on a FACSCalibur cytometer (Beckton Dickinson) and the results were analyzed on FlowJo software (Tree Star, United States).

### Identification, Cloning, and Analysis of the Em*act* cDNA and Gene

The full length sequence of the *Schistosoma mansoni* TGF-β/activin subfamily member (SmInAct, DQ863513) and the human inhibin beta A chain (HsINHßA, P08476) were used to search the *E. multilocularis* genome using the t*blast*n algorithm. Contig 62302 containing coding information for a protein with considerable homology was identified. A predicted gene with a truncated 5′end (EmW_000178100) could be retrieved from the available *E. multilocularis* genome (34). The Full-length coding sequence of the corresponding cDNA was identified by screening of a complementary DNA library (35) and termed *Emact*. Briefly, a consensus sequence between the pathogen_EMU_contig_62302 and Sm*Inact* was used as template for primer design. The following primers were designed and used for retrieval of the 5′ (*Emact*_5′: 5′-ACA GTA GTT GGG TTC-3′) and 3′ (*Emact*_3′: 5′-GAA CCC AAC TAC TGT-3′) ends of Em*act*. These primers were used in pairs with primers specifically recognizing the carrier vector part of the cDNA library, pJG4-5 (35). Once recovered, the 5′and 3′ends of the parasite putative *act* reading frame, were used to design primers for the full length amplification of the *Emact* coding sequence, namely *Emact*_Dw (5′-ATG ACC ATT ACT ACC CCC ATG AAG-3′) and *Emact*_Up (5′-ACT ACA ACC GCA CTC TAG GAC AAT G-3′). Metacestode RNA was isolated using Trizol reagent (Invitrogen) and 1 μg of total RNA was reverse transcribed with Omniscript RT kit (Qiagen) according to the manufacturers′ instructions. The generated cDNA was used as template for amplification of the *Emact* full transcript using the primer pair *Emact*_Dw × *mact*_Up by high fidelity polymerase chain reaction (Phusion, NEB). Resulting amplicons were sub-cloned into the pDrive cloning vector (QIAGEN) and five clones were picked and sequenced in both directions identically revealing the full coding sequence of *Emact* (EmuJ_000178100). Sequence similarities between the deduced amino acid sequence of EmACT and other members of the TGF-β superfamily were determined through multiple sequence alignments using BIOEDIT^1^, and a neighbor-joining tree was generated from alignments using MEGA (36).

### EmACT Antibody Production

For the production of polyclonal antibodies, EmACT was expressed in the bacterial pBAD/TOPO ThioFusion Expression Kit (Invitrogen). To maximize the recognition of EmACT after processing by the generated antibodies, the full *Emact* (without stop codon) amplified using the primer pair *Emact*_Dw/*Emact*_Up coding for the preproprotein EmACT was chosen for immunization and subcloned in pBAD/TOPO ThioFusion expression vector (Invitrogen). The His-tagged Thioredoxin- fusion protein (Thio-EmACT) following arabinose induction (2 g/L; 4 h), was purified on a nickel-nitrilotriacetic acid resin (Invitrogen). The purified protein was diafiltered on against sterile PBS (1×) on Centrifugal Filter Units (Millipore) before quantification. NMRI mice were immunized with the purified Thio-EmACT resuspended in 100 μl of Freund Incomplete adjuvant (Sigma) by two subsequent sub-cutaneous injections initially and repletion 4 weeks later for boosting. Ten days after the second round of injections, animals were terminally bled from the heart and the serum was collected and stored at −20°C. To generate pre-immune serum, naïve NMRI mice were similarly bled by cardiac bleeding and serum collected.

### Recombinant Expression of EmACT in Human Embryonic Kidney Cell Line

Em*act* (full length without signal peptide) was subcloned in the eukaryotic pSecTag2 expression system (Invitrogen) to generate the pSegTag2-Em*act* vector construct as per the manufacturer instructions. Human embryonic kidney cell-line 293T (HEK 293T) were transfected with the expression vector construct pSegTag2-Em*act* or the empty pSecTag2 vector (Invitrogen) as control. Transfections were performed using linear polyethyleneimine (25 kDa, Sigma) according to the manufacturer′s instructions. All transfections were performed in petri dish (92 × 16 mm [Ø x height], SARSTEDT). HEK cells were seeded 16 h prior to transfection (3 × 10^6^ cells/dish). 24 h post-transfection, the supernatants were replaced with fresh DMEM10 medium i.e., DMEM ^+^ GlutamaxTM, (GIBCO) supplemented with 10% FBS Superior (Biochrom AG), 100 μg/ml Penicillin/streptomycin (PenStrep solution, Biochrom AG) and 20 μg/ml Levofloxacin (Tavanic, Sanofi-Aventis, Deutschland GmbH) (thesis). The supernatants of transfected HEK cells were then collected after 24 h of incubation, filtered and normalized for protein content before storage at −80°C until use.

### Immunodetection

To detect EmACT in the supernatants of parasite cultures (natural) or HEK 293 T cell cultures (recombinant), 1 ml of metacestode vesicle E/S products (MVE/S) or pSecTag2-Em*act*-transfected HEK cell supernatant was resuspended in 9 volumes of 100% ice-cold ethanol for protein precipitation as previously defined (thesis). the precipitates were resuspended in 50 μl of 2 × STOPP mix (2 ml 0.5M Tris–HCl pH 6.8, 1.6 ml glycerol, 1.6 ml 20% SDS, 1.4 ml H2O, 0.4 ml 0.05% (w/v) bromophenol blue, 7 μl β-mercaptoethanol per 100 μl) and boiled for 10 min at 100°C. The protein preparations (10μl of each) were then run on a SDS-PAGE, transferred to a nitrocellulose membrane and probed with immune sera for immunodetection.

### Generation of Murine Bone Marrow-Derived Dendritic Cells (BMDC)

Dendritic cells were obtained by 8-day GMCSF-driven differentiation of mice bone marrow precursor cells as previously described (37).

### Isolation of Splenocytes and Lymph Node Cells

Single cell suspensions were obtained from the spleen and lymph nodes of C57Bl/6 mice as previously described (6, thesis, 38). Cell counts were subsequently determined for splenocytes and lymph node cells on a Neubauer counting chamber.

### Treg Conversion Assays

CD4 + T-cells were isolated from murine splenocytes and lymph node cells using a T-cell negative selection kit (Easy Sep mouse T-cell enrichment kit, Stem Cell Technologies) to a purity >90% as per the manufacturer′s instructions. CD25^–^ cells were enriched in CD4 + cells on a MACS separator using LD columns (Miltenyi Biotec). Murine BMDCs were incubated at a ratio of 1:3 with CD4^+^ CD25^–^ T-cells (OT-II or OT-II.RAG-1^–/–^) and OVA peptide (323–339, grade V, 200 ng/ml, Sigma) with different stimuli. In some assays, the cultures were supplemented with 20ug/ml of a pan-vertebrate anti–TGF-β blocking antibody 1D11 (R&D Systems), alongside stimuli addition. In others, isolated naïve T-cells were pre-incubated for 30 min with 5uM of an inhibitor of TGF-β superfamily type I activin receptor-like kinase (ALK) receptors ALK4, ALK5, and ALK7 (SB431542, 42) before cultivation with BMDC.

Alternatively, CD4^+^ T-cells were purified from wild type C57Bl/6 mice and subsequently activated on plate-bound CD3 (1 μg/ml) and CD28 (0.5 μg/ml) antibodies in the absence or presence of our different stimuli. Recombinant human TGF-β1 (R&D Systems) was used as positive control. After 5 days of incubation, the cells were collected and stained for acquisition on a FACSCalibur cytometer (Beckton Dickinson) and the results analyzed on FlowJo software (Tree Star, United States).

### CD4^+^ T-Cells Stimulation Assay

Single CD4^+^ T-cell suspensions from spleens and lymph nodes of C57Bl/6 mice (6–8 weeks old) enriched for CD25^–^ cells were seeded as 2 × 10^5^ cells per well in a CD3 antibody (0.1 μg/ml, eBioscience) pre-coated 24-well tissue culture plate (Flat bottom, SARSTEDT) with CD28 antibody (5 μg/ml, eBioscience) and different stimuli. After 72 h, the culture supernatants were probed for IL-10 release by ELISA with a detection limit of 19 pg/ml (BD OptEIA^TM^ – Mouse IL-10 ELISA Set – BD Biosciences).

### Statistical Analyses

All results were expressed as mean ± standard deviation (SD). Differences were evaluated between two groups using the Wilcoxon/Mann-Whitney *U* test or Wilcoxon Signed-Rank Test, non-parametric tests that do not assume normality of the measurements (they compare medians instead of means). *p* < 0.05 defined statistical significance and statistical analyses were done with GraphPad Software.

### Accession Numbers

The complete *Emact* cDNA sequence reported in this paper was deposited in the GenBank database under the accession number HF912278. All GenBank accession numbers of sequences and genes used in this study are listed in Supplementary Table S1.

## Results

### *E. multilocularis* Metacestode Tissue Drives Foxp3^+^ Treg Expansion in Experimentally Infected Mice

In previous reports, it has been shown that Treg are expanded during chronic secondary AE 6–12 weeks post infection (15, 28). It has not been elucidated, however, whether this expansion resulted from an inherent host protective mechanism in the course of a chronic infection in order to minimize tissue damage, or whether Treg expansion was actively driven by the parasite. Since chronic AE, other than early AE, is associated with severe depletion of T-cells after 6 weeks of infection (38), we investigated the dynamics of host CD4^+^ T-cell responses during experimental secondary AE for up to 7 weeks (42 days) post infection; i.e., up to the chronic phase of the disease. To this end, we injected mice intraperitoneally with 5000 *E. multilocularis* acephalic cysts (metacestode), axenically sub-cultured for up to 10 days to remove host cells (as confirmed by species-specific PCR, see Supplementary Figure S1), and analyzed the peritoneal exudate cells over a 7-weeks period (42 days).

**FIGURE 1:**
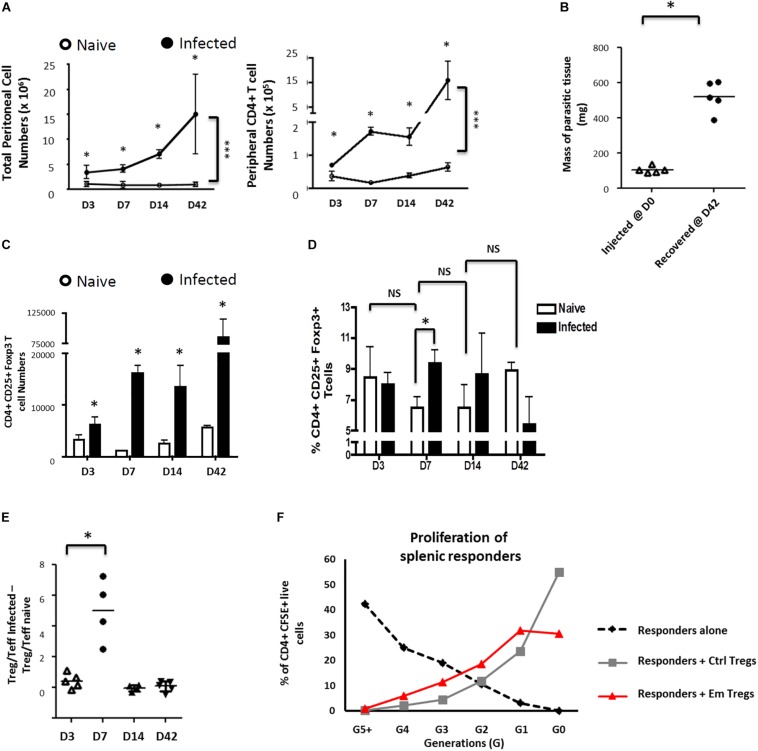
*Echinococcus multilocularis* metacestodes expand functionally suppressive Foxp3 + T regulatory T cells *in vivo*. Peritoneal exudate cells from control and *Em*-injected animals were collected, counted, and analyzed by flow cytometry for CD4 expression. **(A)** Parasite-driven accumulation of total (left) or CD4^+^ T-cells (right) is shown for D3-42 post injection. **(B)** Masses of parasitic tissue injected and recovered after 42 days. **(C)** The kinetics of total Foxp3^+^ Treg numbers was monitored. The peritoneal exudate cells recovered were analyzed by flow cytometric analysis for CD25 and Foxp3 expression. **(D)** The kinetics of Foxp3^+^ Treg frequencies (as percentages of total peritoneal CD4 + T cells) was monitored. **(E)** Kinetics of Treg/Teff ratio over time as a measure of the bias of parasite-associated CD4^+^ T-cell response (where percentages of total CD4 + T cells that are CD25 + Foxp3 + represent Treg frequencies and percentages of total CD4 + T cells that are CD25 + Foxp3 – represent Teff frequencies). Each ratio for infected mice was substracted of the corresponding naive mice ratio. **(A–E)** *X*-axis represent days after infection. Data represent means ± SD from groups of five mice for each time point assayed individually (Infected). Naive mice were clustered in sub-groups of 3 mice pooled as one per assay (15 mice for each time point). Data were compared using Mann–Whitney *U* test ^∗^*p* < 0.05. NS, not significant. **(F)** Peritoneal exudate cells from 15 mice infected for 7 days with 5000 acephalic *E. multilocularis* cysts each, and naive splenocytes from control mice, were prepared by CD4^+^ T-cell magnetic selection, then FACS-sorted into CD4^+^CD25^+^ and CD4^+^CD25^–^ populations. Splenic naive CD4^+^CD25^–^ cells (responders) were then polyclonally stimulated in the presence or absence of CD4^+^CD25^+^ cells from either control or *E. multilocularis*-infected mice. Proportions of labeled live CD4^+^ cells in each generation of assay conducted at 1:1 ratio, as gated by CFSE dilution. A representative experiment out of two cell preparations with similar results is displayed.

We observed a significant increase of peritoneal exudate total and CD4^+^ T-cells over time in *E. multilocularis* infected mice as compared to mock (PBS)-injected controls (Figure 1A). Interestingly, despite the anti-AE role of host cellular immunity in general and CD4^+^ T-cell mediated effector functions in particular (39), the CD4^+^ T-cell expansion in infected mice was paralleled by an increase in parasite mass during the study period (Figure 1B).

We then examined the subsets of CD4^+^ T-cells expanded in infected mice by expression levels of CD25 and Foxp3 (validation of staining as defined in Supplementary Figure S2A). A separation into CD25^+^Foxp3^–^ CD4^+^ activated effector T-cells (Teffs) and CD25^+^Foxp3^+^ CD4^+^ T-cells as Tregs was applied (Supplementary Figure S2B). Although we noted a general increase of Foxp3^+^ Treg numbers in infected mice when compared to naïve mice throughout the study period (Figure 1C), a transient but significant increase of the proportion of Tregs was uniquely detectable at 7 days post-infection within the peritoneal exudates of mice (Figures 1D,E). The Tregs induced by the parasite 7 days post intraperitoneal inoculation were able to repress proliferative responses of CFSE-labeled conventional CD4^+^CD25^–^ T-cells (Figure 1E), indicating that they were functionally suppressive.

Taken together these analyses showed that *E. multilocularis* metacestodes can grow in these mice and raise a CD4^+^ T-cell response but with a transient overproportional expansion of suppressive Tregs.

### E/S Products of the *E. multilocularis* Metacestode Induce Foxp3 Expression and IL-10 Production by Host T-Cells *in vitro* and Host TGF-β and TGF-β Signaling Are Essential for Treg Conversion Driven by Metacestode E/S Products

**FIGURE 2:**
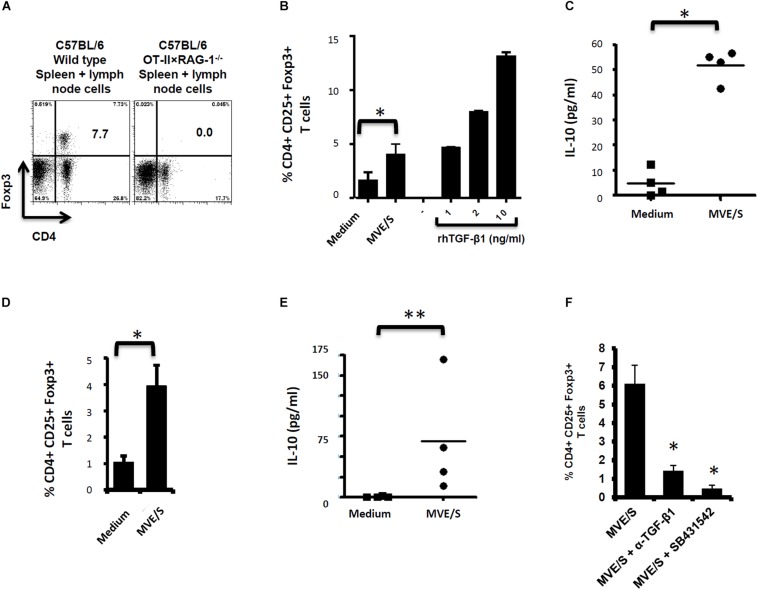
E/S products of *E. multilocularis* metacestode promote the *de novo* Foxp3^+^ Treg conversion and IL-10 production by naïve T cells *in vitro*. **(A)** Staining of CD4^+^ Foxp3^+^ T-cell within the bulk of spleen and lymph node cells from wild type C57Bl/6 or C57Bl/6 OT-II.RAG-1^–/–^ mice over a C57Bl/6 background. This shows the lack of Foxp3 + cells among CD4 + T cells when isolated from the lymphoid organs of naïve C57Bl/6 OT-II.RAG-1^–/–^ mice. **(B)** MVE/S promote *de novo* CD4^+^CD25^+^Foxp3^+^ Treg conversion *in vitro*. Freshly generated DCs (Day 8, from 3 bone marrow cell preparations) were co-cultured individually with 3 cell preparations of naïve CD4^+^CD25- T-cells from 3 OT-II.RAG-1^–^/^–^ mice at a DC:T-cell ratio of 1:3 in R10 medium supplemented with OVA peptide (200 ng/ml). E/S-free serum-supplemented medium (DMEM10 redox) or MVE/S-containing (DMEM10 redox) medium was added to the cultures prior to incubation. Different doses of recombinant human TGF-β1 were used as positive controls. 5 days later, cells were harvested and stained for CD4, CD25 and Foxp3 prior to flow cytometry analysis. **(C)** Additionally, culture supernatants were collected and probed for IL-10 by ELISA. **(B,C)** Summarized in the graph are the percentages of CD25^+^ Foxp3^+^ cells within the CD4^+^ T-cell population and the production of IL-10 measured after exposure to the indicated stimuli. Data represent mean ± SD from two independent experiments with products from two different parasite isolates. **(D)** Foxp3^+^ Treg frequencies in CD4^+^ T cells cultured for 5 days on CD3/CD28 antibody-coated plates in the presence of E/S-free medium (DMEM10 redox) or MVE/S-containing medium. Bars represent the mean ± SD of results obtained with E/S products from 4 different parasite isolates tested in 2 independent experiments on fresh T-cell preparations. ^∗^*p* < 0.05. **(E)** Naïve CD4^+^ CD25^–^ T-cells freshly isolated from C57Bl/6 mice were stimulated at 2 × 10^5^/ml with CD3/CD28 antibodies in the presence of parasite E/S-free cultivation medium (DMEM10redox) or MVE/S-containing (DMEM10 redox) medium. After 72 h, the T-cells supernatants were collected and probed for IL-10 concentration by Elisa. Horizontal bars represent the mean from experiments conducted with E/S products from 4 different parasite isolates tested in 2 independent experiments on fresh T-cell preparations. Data were compared using Mann–Whitney *U* test ^∗^*p* < 0.05. ^∗^*p* < 0.05; ^∗∗^*p* < 0.005. **(F)** Blocking TGF-β signaling or host TGF-β alone abrogates *E. multilocularis*-driven Treg conversion *in vitro*. Mean percentages of Foxp3^+^ Treg within the CD4^+^ T-cell population of OT-II naïve CD4^+^ T-cells cultivated with freshly generated DC (Day 8) at a DC:T-cell ratio of 1:3 in R10 medium supplemented with OVA peptide (200 ng/ml) in the presence of MVE/S-containing medium alone (supplemented with DMSO in one out of two experiments), combination of MVE/S-containing medium with TGF-β antibody or combination of MVE/S-containing medium with SB431542 (resuspended in DMSO). Flow cytometry was performed 5 days later. Bars represent mean ± SD from two independent experiments with fresh DC/T cell preparation in each experiment. Data were compared using Mann–Whitney *U* test ^∗^*p* < 0.05.

We previously demonstrated Foxp3^+^ Treg expansion *in vitro* from OT-II naïve CD4^+^ T-cells activated with OVA-loaded DC in the presence of *E multilocularis* MVE/S (6), suggesting either a direct induction of Treg conversion by the parasite products or a mitogenic effect of these products on pre-existing OT-II Treg. To further examine these alternatives, we isolated naïve OT-II.RAG-1^–/–^ CD4^+^T-cells from spleens and lymph nodes of naïve animals, genetically devoid of Foxp3^+^ Tregs (Figure 2A). The cells were activated *in vitro* with OVA-loaded DCs in the presence of *E. multilocularis* MVE/S as previously described (6). MVE/S failed to activate BMDC cultures beyond the baseline level obtained with medium, arguing against a potential contamination of the harvested parasite products with endotoxins (6). Notably, Foxp3^+^ Treg frequencies were considerably enhanced in cultures supplemented with MVE/S, similar to TGF-β (Figure 2B), suggesting that *E. multilocularis* MVE/S can induce *de novo* Foxp3^+^ Treg conversion from naive CD4^+^ T cells *in vitro*. We also measured the production of the immunosuppressive cytokine IL-10 in DC-T-cell co-cultures in the presence or absence of the parasite products. We noted a significantly increased production of IL-10 in cultures supplemented with *E. multilocularis* metacestode products (Figure 2C) indicating that the parasite products can both expand host Foxp3^+^ Treg, and also trigger an elevated production of IL-10 by host immune cells.

Next, to investigate the role of the DC population in T-cell modulation by *E. multilocularis* products, naïve CD4^+^ T-cells from spleens of C57Bl/6 mice activated with plate-bound anti-CD3 and anti-CD28 antibodies (instead of DC-based activation) in the presence of *E. multilocularis* MVE/S were both analyzed for Foxp3 expression and IL-10 production (see section “Materials and Methods” for experimental set-ups). We observed an increased rate of Foxp3^+^ Treg (Figure 2D), and a significantly elevated production of IL-10 (Figure 2E) in host T-cell cultures indicating that *E multilocularis* metacestode products can induce Foxp3^+^ Treg conversion and trigger IL-10 release by naïve T-cells in a DC-independent manner.

As it has previously been shown that the conversion of CD4^+^ T-cells to Treg requires TGF-β (40), we could not exclude that the complex, serum-containing media required for parasite cultivation do contain this cytokine to a certain amount. To analyze whether MVE/S require host TGF-β activity to promote Treg conversion, anti-TGF-β neutralizing antibodies were used. The performed assay showed a clear inhibition of Foxp3^+^ Treg conversion by MVE/S when TGF-β was neutralized (Figure 2F). To additionally confirm an important role of TGF-β in the ability of metacestode products to expand host Treg, the TGF-β signaling inhibitor SB431542 (41) was used. Again, we observed a drastic reduction of the rate of Foxp3^+^ Treg induced by MVE/S (Figure 2F). Taken together, we conclude from these studies that MVE/S can induce the conversion of naive CD4^+^ T-cells into Foxp3^+^ Treg *in vitro* and that this activity depended on the presence of host TGF-β and functional TGF-β signaling in host cells.

### *E. multilocularis* Expresses an Activin A – Like Cytokine

By literature search for molecules that could exert activities as observed above for metacestode E/S products, we found striking similarities to the mammalian TGF-β-like cytokine activin A. Like metacestode E/S products, activin A can induce Treg conversion *in vitro*, which depends on host TGF-β and functional TGF-β signaling (42, 43), and it can induce the secretion of IL-10 by CD4^+^ T-cells (43). Interestingly, the expression of activin-like cytokines, SmInAct (44) and FhTLM (45), have previously been reported for the related flatworm parasites *Schistosoma mansoni* and *Fasciola hepatica*, respectively. Both of these molecules influence parasite development and immunoregulatory functions have also been demonstrated for the latter (46). We therefore hypothesized that *E. multilocularis* might also express an activin A-like molecule and performed extensive BLASTP analyses on the published genome sequence (34), using mammalian inhibin beta A (activin A monomer) and SmInAct as queries. These analyses revealed the presence of one single-copy gene, EmuJ_000178100, encoding a protein with significant homologies to both query sequences. Since further genome mining did not yield indications for the presence of additional inhibin/activin-encoding genes, implying that the cytokine encoded by EmuJ_000178100 can only form homo- but not heterodimers, the respective gene was designated *Emact* (for *E. multilocularis* activin) and registered under the Genbank accession number HF912278.1.

**FIGURE 3:**
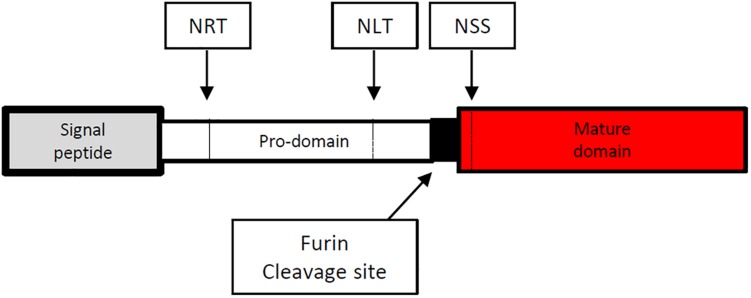
Diagrammatic representation of the amino acid sequence of the *Echinococcus multilocularis act* protein-coding sequence. The 5′signal sequence is shown at the left end in gray spanning amino acid (aa) 1–29. The potential prodomain spanning aa 30–374 is shown as an open box followed at the right by a paired dibasic furin cleavage motif (RTRR) in black. The C-terminal end is composed of a TGF-β superfamily active domain (aa 378–507) shown in red. N-glycosylation sites (NRT, NLT and NSS) are shown in dashed vertical lines.

**FIGURE 4:**
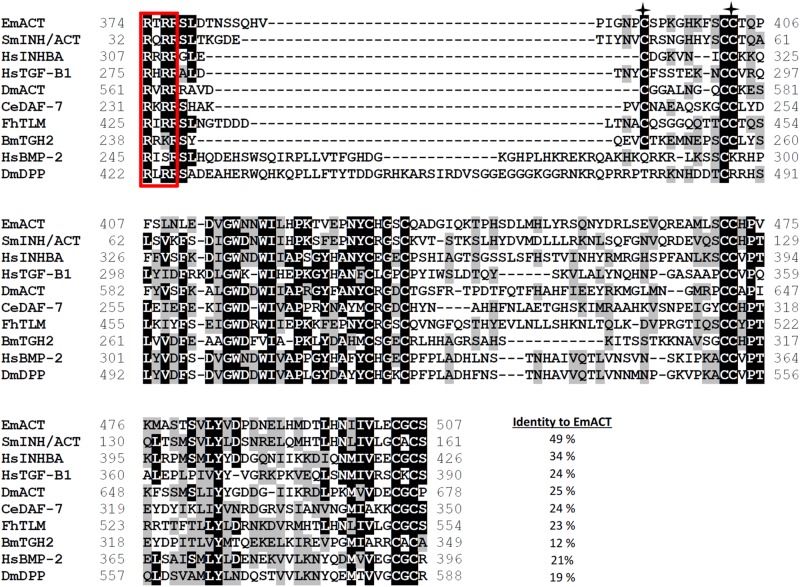
Alignment of the C-terminal amino acid sequences of EmACT and seven other representatives of the TGF-β superfamily. The paired dibasic cleavage motif is shown within a red open box. Residues that are identical are highlighted in black, similarities in gray. Gaps introduced to maximize the alignment are represented by dashes. Two conserved cysteines found only in TGF-β/activin subfamily are shown with asterisks. Numbers at the start and finish of each line correspond to the amino acid numbers in each respective sequence. Accession numbers for the sequences shown are as follows: *Echinococcus multilocularis act*, HF912278; *Schistosoma mansoni* InAct, A4UAH0; Human Inhibin beta A, P08476; Human TGF-β 1, P01137; *Drosophila melanogaster* Activin, O61643; *Caenorhabditis elegans* DAF-7, P92172; *Fasciola hepatica* TGF-like Molecule FhTLM; *Brugia malayi* TGF-beta homolog, BmTGH2, AAD19903.1; Human BMP-2, P12643; and *Drosophila melanogaster* DPP, P07713.

The full length cDNA of *Emact* was cloned and sequenced and comprised 1536 bp that encoded a 507 aa protein, EmACT, with a hydrophobic region at the N-terminus, indicating the presence of an export-directing signal peptide (Figure 3). Structurally, EmACT displayed several conserved features of the TGF-β cytokine superfamily such as a C-terminal, cysteine-rich active domain, separated by a tetrabasic RTRR cleavage motif from a large N-terminal and less well conserved pre-protein sequence. Within the C-terminal active domain of all TGF-β superfamily members (activins and BMPs) are seven invariant cysteine residues, six of which form a rigid, heat stable “cysteine knot” (23). Accordingly, the C-terminal domain (130 aa) of EmACT contained all these invariant cysteines (Figures 3, 4), as well as two additional cysteines (Figures 3, 4) that are characteristic of TGF-β/activin subfamily members, but that are not present in BMP subfamily members (Figure 4).

**FIGURE 5:**
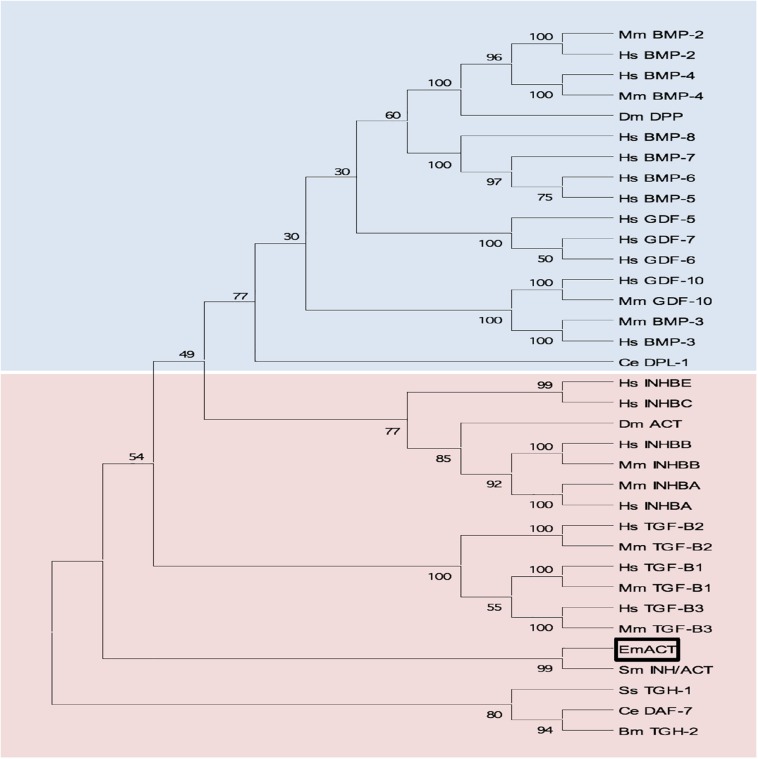
Phylogenetic clustering of EmACT with TGF-β/activin subfamily members. A non-redundant set of TGF-β superfamily members sequences were aligned and an unrooted neighbor-joining tree was computed by MEGA. EmACT is shown clustering with members of the TGF-β/activin subfamily (pink box), but not with members of the BMP/growth differentiation factor subfamily (blue box). Conserved residues in the C-terminal region of each homolog (final 94–106 amino acids) were used in the analysis. Percentages at branch points are based on 1,000 bootstrap runs.

Sequence comparisons to several TGF-β superfamily members (Figure 4) and BLASTP analyses of the conserved C-terminal portion of EmACT against protein databases confirmed that its closest relatives are inhibin beta A chains. Highest homologies were detected to SmInAct (49% identical amino acid residues) and human inhibin beta A (34%) (Figure 4). To further confirm that EmACT is an activin/inhibin ortholog, we carried out phylogenetic analyses. The putatively bioactive C-terminal domain of EmACT was aligned to those of several TGF-β superfamily members and the degree of homology was represented on a phylogenetic tree. EmACT clearly clustered with TGF-β/activin subfamily members but not with the BMP subfamily (Figure 5) and, again, showed highest similarity to SmInAct. Taken together, all structural analyses clearly indicated that EmACT is a member of the TGF-β/activin subfamily of TGF-β like cytokines.

Finally, by using the EmACT sequence as a query in BLASTP analyses against the recently determined genome sequences of other cestodes (34), we identified *Emact* orthologs in *E. granulosus* (EgrG_000178100), *Taenia solium* (TsM_000011500), and *Hymenolepis microstoma* (HmN_000204000), which encoded proteins with 99, 95 and 66% amino acid sequence identity to EmACT, respectively. Hence, the presence of activin A – encoding genes appears to be a common feature of tapeworm genomes.

### Heterologous Expression of EmACT

**FIGURE 6:**
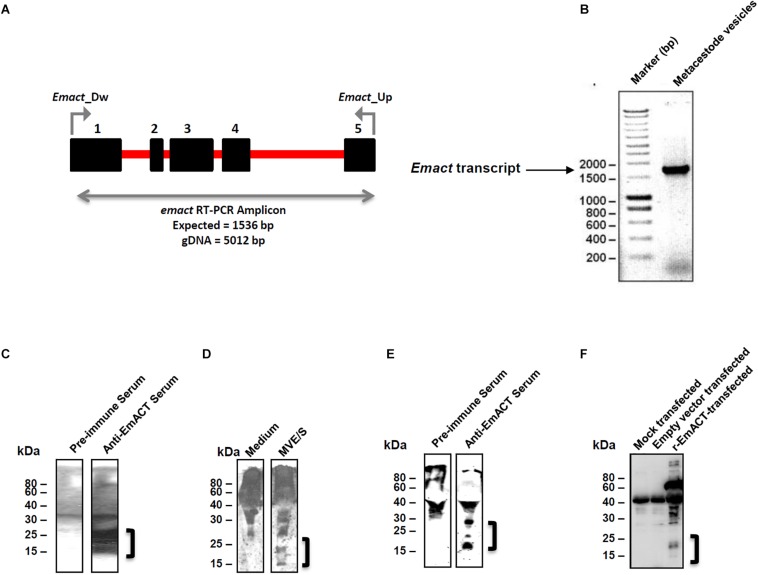
Detection of EmACT. **(A)** RT-PCR strategy for unequivocal amplification of *Emact* transcript. Shown is an intron (red line)-exon (black boxes) arrangement of the E*mact* genomic locus. A 1536 bp product for *Emact* full transcript was amplified using the primers E*mact_Dw* and *Emact_Up* spanning from exons 1–5. **(B)** *E. multilocularis* MV were used for qualitative assessment of *Emact* expression. 1 μl of larvae cDNA was used as template for PCR with a high fidelity DNA polymerase (Phusion High-Fidelity DNA Polymerase, New England Biolabs). 2 μl of PCR amplicon were resolved on a 1.5% agarose gel and stained with Ethidium bromide prior to visualization under a UV transilluminator. *Emact* was then cloned into the bacterial expression vector pBADThio/TOPO. Competent *E.coli* (Top 10) bacteria were transformed with the Thio-Em*act* plasmid and induced to express the fusion Thio-EmACT protein under arabinose control. A C-terminal histidine repeats fused to the expressed Thio-EmACT fusion protein by the pBADThio/TOPO expression vector was used as target tag for protein purification over Nickel-supplemented beads and the purified full-length, inactive Thio-EmACT was injected into mice to generate anti-EmACT immunserum. **(C)** Secretion of EmACT by *Echinococcus multilocularis* metacestode vesicles in culture. Shown is a western blotting of ethanol-precipitated MVE/S probed with normal mouse serum or mouse anti-EmACT Immunserum followed by ECL detection and autoradiography. The positions of the molecular mass markers (in kilodaltons) are shown on the left. The bracket indicates the position of EmACT variants. **(D)** Mouse anti-EmACT Immunserum probing of the Ethanol-precipitated parasite-conditioned medium and naive culture medium. The positions of the molecular mass markers (in kilodaltons) are shown on the left. The bracket delimitates the location of recombinant EmACT variants. **(E)** Secretion of recombinant EmACT by pSecTag2-*emact*-transfected HEK cells. The vector construct used to transfect HEK cells contains the full length E*mact* coding sequence (minus the original signal peptide). Shown is a western blotting of the Ethanol-precipitated supernatant of pSecTag2-*emact-* transfected 293T HEK cells probed with either normal mouse serum (or mouse anti-EmACT immune serum followed by ECL detection and autoradiography. The positions of the molecular mass markers (in kilodaltons) are shown on the left. The bracket delimitates the location of recombinant EmACT variants. **(F)** Mouse anti-EmACT Immunserum detection of rEmACT in the Ethanol-precipitated supernatant of pSecTag2-*emact*-transfected HEK cells. Shown is a western blotting of the Ethanol-precipitated supernatant of mock, pSecTag2- or pSecTag2-*emact-* transfected 293T HEK cells probed with mouse anti-EmACT immune serum followed by ECL detection and autoradiography. The positions of the molecular mass markers (in kilodaltons) are shown on the left. The bracket delimitates the location of recombinant EmACT variants.

Preliminary deep sequencing transcriptome data collected during the *E. multilocularis* whole genome sequencing project (34) indicated that *Emact* is actively transcribed in the *E. multilocularis* metacestode. We validated this on metacestode vesicles cDNA by using an emact specific reverse transcription PCR scheme (Figure 6A). The *Emact* full transcript of 1536bp was successfully amplified from metacestode vesicle cDNA (Figure 6B) confirming that the parasite expresses this factor. This was also the case when using either primary cells or protoscoleces cDNA arguing for a general expression of the factor by all larval stages (Supplementary Figure S3). To closely investigate the formation of the gene product, EmACT, an anti-EmACT antiserum was raised in mice by subcutaneous injection of tag-fused EmACT. This polyclonal serum was used to specifically assess whether EmACT is secreted by *E. multilocularis*. The supernatant of *in vitro* cultivated metacestode vesicles was probed with the anti-EmACT antiserum. We clearly detected reactive proteins of 15- to 25-kDA in the supernatant (Figures 6C,D), indicating that EmACT is secreted by *E. multilocularis* metacestodes and is processed into glycosylated or non-glycosylated forms of a single homodimer of ∼25 kDa, or monomer of 12.5 kDa, consistent with the varieties of processing options for TGF-beta family members.

For functional characterization of EmACT, the entire protein-coding region was recombinantly expressed in HEK 293 cells under the control of the cytomegalovirus promoter using a mammalian expression system. As shown by Western blotting using the anti-EmACT antiserum, recombinant EmACT (rEmACT) was secreted to the medium by transfected HEK 293 cells as 15- to 25-kDa variants (Figures 6E,F), which was in agreement with the observed secretion pattern of mature EmACT by the *E. multilocularis* metacestode (Figures 6C,D). This indicates the ability of HEK cells to use endogenous furin to cleave EmACT preprotein. To confirm the processing and secretion of EmACT in our heterologous system, HEK 293 cells were transfected with full length EmACT (without the original signal peptide) with a myc tag inserted within the coding sequence, after the furin cleavage site and prior to the mature peptide (Supplementary Figure S4) to conceptually enable the secretion of a N-tagged mature EmACT in culture. This was carried in order to validate that the assumed complex processing typical of TGF-β superfamily is relevant in EmACT. Indeed, the secretion of c-myc-N-tagged mature EmACT was assessed by immunoprecipitation of the transfected HEK cell supernatant using bead-bound anti-c-myc antibodies and probing the beads’ eluate with anti-c-myc antibodies, revealing specific bands by around 15- to 25-kDa (Supplementary Figure S4).

Collectively these results showed that EmACT is secreted by *E. multilocularis* metacestodes as differentially processed variants, which could also be efficiently produced by recombinant expression of EmACT in HEK cells.

### rEmACT Induces Treg Conversion *in vitro*

**FIGURE 7:**
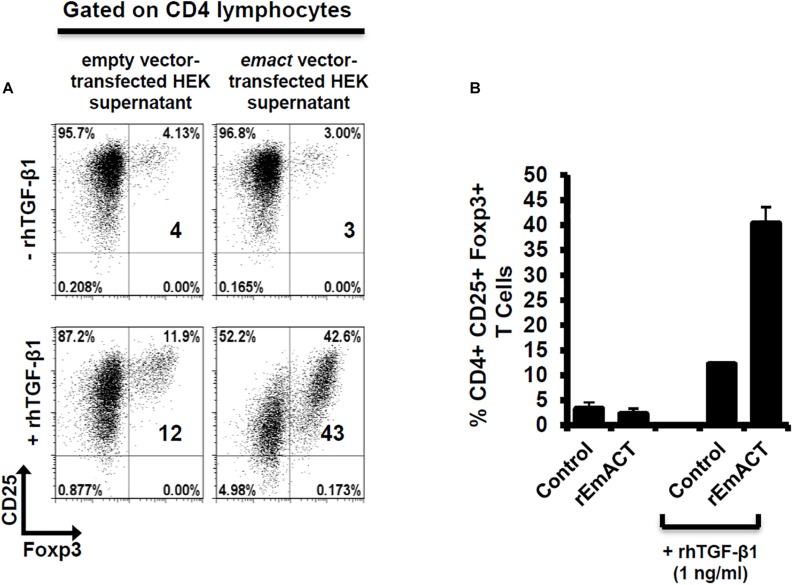
EmACT promotes host TGF-beta-dependent Foxp3^+^ Treg conversion *in vitro*. Freshly generated BMDCs (Day 8) were co-cultured with naïve (CD25^–^) OT-II.RAG-1^–^/^–^ CD4^+^ T-cells at a DC:T-cell ratio of 1:3 in R10 medium supplemented with OVA peptide (200 ng/ml) in the presence of supernatant from pSecTag2-transfected HEK (Control) or pSecTag2-*emact*-transfected HEK (rEmACT) supplemented or not with rhTGF-β1 (1 ng/ml). After 5 days of incubation, cells were harvested and stained for CD4, CD25 and Foxp3 prior to flow cytometry analysis. **(A)** Representative plots of two independently performed Treg conversion assays with two different DC/T cell preparations with supernatant from 2 batches of transfected HEK cells summarized in **(B)**. The bars represent the mean ± SD.

Similar to our previous assays using metacestode E/S products, we investigated whether rEmACT has activin A–like activities. Again, purified naïve CD4^+^CD25^–^ T-cells from spleens and lymph nodes of OT-II.Rag-1^–/–^ mice were isolated and co-cultured with OVA-pulsed DCs. The supernatant of *Emact*-transfected HEK cells (rEmACT) or vector-transfected HEK 293 cells (control) were added to the DC-T-cell co-cultures and the rate of Foxp3^+^ Treg conversion was measured 5 days later by flow cytometry. When compared to the supernatant of vector-transfected HEK 293 cells, rEmACT-containing HEK cell supernatant alone failed to expand Foxp3^+^ Treg but could considerably promoted TGF-β-driven Foxp3^+^ Treg conversion (Figure 7). These data indicated that EmACT is unable to induce the *de novo* Foxp3^+^ Treg conversion alone, but synergizes with TGF-β.

### rEmACT Induces the Secretion of IL-10 by CD4^+^ T-Cells *in vitro*

**FIGURE 8:**
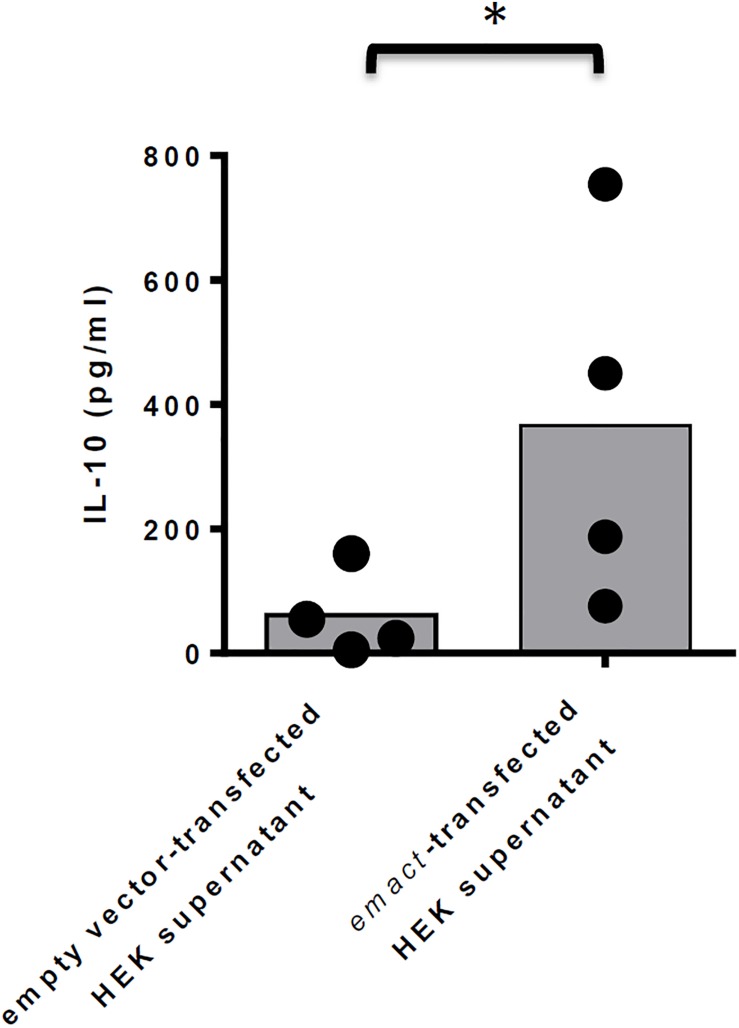
EmACT promotes IL-10 release by CD4^+^ T-cells *in vitro*. CD4^+^CD25^–^ T-cells freshly isolated from C57BL/6 mice were stimulated with CD3/CD28 antibodies in the presence of supernatants from pSecTag2-transfected (Control) or pSecTag2-*emact*-transfected HEK cells (rEmACT). After 72 h, the T-cells supernatants were collected and probed for IL-10 concentration by Elisa. Horizontal bars represent the mean from two independent experiments with T-cells from two different isolations individually activated in the presence of HEK supernatant batches from two different transfections. *p < 0.05.

Finally, we also investigated whether rEmACT, like mammalian activin A, is able to stimulate the secretion of IL-10 by CD4^+^ T-cells. Naïve CD4^+^ CD25^–^ T-cells from spleens of C57Bl/6 mice were activated with plate-bound anti-CD3 and anti-CD28 antibodies and the supernatants of *Emact*- or vector-transfected HEK 293 cells were added as test and control, respectively. We noted a considerably higher production of IL-10 in T-cell cultures supplemented with rEmACT-containing HEK supernatant when compared to the control (Figure 8) suggesting that rEmACT can trigger IL-10 release by CD4^+^ T-cells *in vitro*.

## Discussion

Alveolar echinococcosis is a chronic disease characterized by continuous and infiltrative (tumor-like) growth of the *E. multilocularis* metacestode stage over years or even decades within the organs of the intermediate host [1,2,5]. Previous work established that this is associated with considerable immune suppression, provoked by parasite surface structures and metabolites that are released by the actively growing metacestode (1, 5, 6, 8, 9, 13–17, 28, 33, 38, 47). In other long-lasting helminth infections, regulatory T-cells had been identified as major contributors to parasite-induced immune suppression (20, 32, 48–50) and, very recently, evidence for the expansion of this cell type during secondary AE has been obtained (15, 28) with a critical role demonstrated for their immunosuppressive functions in the mitigation of host anti-AE immune response (28). However, it was not clear from these studies whether Tregs were actively induced by the parasite *in vivo*. Evidence for a certain capacity of *Echinococcus* E/S products to induce Tregs was recently obtained by us in a DC-based Treg expansion assay (6). This study did not discriminate, however, between mitogenic effects on pre-existing Tregs and *de novo* Treg conversion. Furthermore, the precise mechanism of Treg expansion by *E. multilocularis* remained elusive.

Our present results indicate an active induction of Treg by the parasite as follows: (i) Using an *in vivo* infection model for secondary AE (8, 14–16, 38, 39, 47), we observed a significant expansion of Foxp3 + Treg over effector T-cells in the peritoneum of mice in a time window around 7 days post infection. (ii) We demonstrate that Treg formed at this time point are functionally suppressive. (iii) Rather than inducing the proliferation of pre-existing Treg, MVE/S can efficiently promote the *de novo* conversion of host Treg *in vitro*, in a TGF-β-dependent manner. (iv) In addition to inducing tolerogenic phenotypes in T-cell-priming DC (6), MVE/S can also promote host Treg conversion in a direct, DC-independent manner. Taken together, these data clearly indicate that Foxp3^+^ Tregs can be actively induced by the *E. multilocularis* metacestode to drive host immune suppression during AE.

Interestingly, we also found that MVE/S induce IL-10 release by CD4^+^ T-cells. Whether IL-10 in our assays is produced by Foxp3^–^ or Foxp3^+^ CD4^+^ T-cells is not fully clear at this point. However, since the CD4^+^ T-cell-dependent production of IL-10 in response to parasite E/S products clearly preceded the expansion of Foxp3^+^ Treg (3 days vs. 5 days), Foxp3^–^ cells are likely to be the main source of CD4^+^ T-cell-derived IL-10 in our assays, which suggests that the parasite-driven Treg expansion and the induction of IL-10 producing T-cells occur largely independently from each other during AE. This is consistent with previous studies on products of the related trematodes *Schistosoma mansoni* (51) and *Fasciola hepatica* (52) for which also Treg expansion and elevated CD4^+^ T-cell dependent IL-10 production had been observed. Hence, such an independent induction of IL-10 producing T-cells would add to the immunosuppression by Foxp3 + Treg and could further contribute to parasite establishment. It also provides for the first time a mechanistic explanation for the elevated IL-10 levels observed in tissues and body fluids of AE patients (9, 53, 54). However, our present data do not unequivocally ascribe IL-10 production to Foxp3 + Treg cells. The in-depth characterization of IL-10 producing cells elicited by the parasite secretions and products would warrant comprehensive flow cytometric typing of these CD4 + T cells coupled with intracellular staining of IL-10 to identify the CD4 + Tcell subset(s) responsible for the increased IL-10 observed.

Within the *E. multilocularis* metacestode E/S fraction we identified a component, EmACT, that likely contributes to the Treg expansion and the induction of IL-10 secretion by T-cells. Like metacestode E/S products, solutions containing recombinantly expressed EmACT promoted Treg conversion *in vitro* and required host TGF-β to do so. Furthermore, rEmACT-containing solutions also triggered the release of IL-10 by host T-cells. We cannot completely rule out, however, that the metacestode E/S fraction also contains additional factors that contribute to the observed induction of IL-10 by T-cells and/or to Treg conversion. The dependency on host TGF-β, as observed in our settings, suggests an accessory, rather than central, role of this factor in the observed ability of *E. multilocularis* metacestode to expand host Treg. Clearly, other unidentified *E. multilocularis* factors might possess the Treg inducing ability herein reported and in so doing, possibly act in concert with EmACT to promote immunoregulation. In this regard, the *E. multilocularis* genome (34) does, for example, encode homologs of the schistosome ribonuclease omega-1 (55, 56) or mammalian BMPs, which have the ability to induce Treg conversion in a TGF-β-dependent manner (57, 58). However, unlike EmACT, these factors have not been reported to induce IL-10 production in T-cells. To further investigate this aspect, we already tried to block EmACT activities in the E/S fraction by using the available anti-EmACT antiserum. Unfortunately, several attempts to immunoprecipitate native EmACT from E/S products using our generated serum failed, indicating that the available antibodies might only recognize the mature protein in its denatured form. To investigate whether additional metacestode E/S components are capable of inducing Treg conversion and/or IL-10 production by T-cells the availability of neutralizing antibodies that recognize native EmACT would thus be necessary. Nevertheless, even if additional parasite components could contribute to the immunosuppressive activities of the metacestode E/S fraction, our experiments on recombinantly expressed EmACT suggest that it is one component of the cascade of events that promote a Treg and IL-10 rich environment during AE.

In an important previous contribution, Grainger *et al.* (49) demonstrated that E/S products of the nematode *Heligmosomoides polygyrus* can induce Treg *de novo* and suggested a “TGF-β mimic” as the major E/S component to mediate these effects. Although the precise molecule has now been identified in this study as a non-TGF-β superfamily member (59), these authors demonstrated that their molecule acted via the host TGF-β signaling cascade to mediate its effect. In fact, identified nematode TGF-β orthologs also have the capacity to bind to mammalian TGF-β receptors (60). We here show that a helminth-derived TGF-β-superfamily member can promote Treg (TGF-β-dependent) and, at least concerning immune cells, displays clear functional homologies to activin A such as the induction of IL-10 in T-cells (42, 43). Interestingly, our *in silico* analyses also identified similar activin-like molecules in the genomes of other cestodes. Notably, *E. granulosus*, *Taenia solium* and *Hymenolepis sp.* which are pathogens reported to expand Foxp3^+^ Treg and elevated IL-10 production in their hosts (61–65), do all harbor *Emact* orthologs. An implication of this family of molecules in the modulation of the host immune response by these related helminths is therefore possible and merits closer examination.

The fact that E/S products from *E. multilocularis* metacestodes can induce IL-10-secreting and Foxp3^+^ T-cells, which themselves might produce or convey to other immune cells the ability to produce immunosuppressive cytokines like TGF-β and IL-10 (66, 67), could explain the high doses of these cytokines found in parasite vicinity during AE infections (10, 11, 53). This tightly reconciles with the reported expansion of CD4^+^ Tregs within the periparasitic environment during AE (15, 28) and the debilitating role of this cell type on the host ability to control the infection (28). Since these granuloma also contain CD8^+^ T-cells (10, 11, 53) we cannot exclude that immunosuppressory mechanisms associated with suppressive CD8^+^ T-cells (15) are also at work. However, since it has been shown that the CD4^+^ fraction is highly important for parasite clearance (39), we think that CD4^+^ Tregs are major actors in the impairment of host immunity during AE. Experiments to further verify this have been performed (28–30) supporting a critical role of this parasite-driven modulation of cell-mediated immunity by Tregs during AE.

Due to the relatively close phylogenetic relationship between helminths and mammalian hosts, it is now clear that they can communicate via evolutionarily conserved signaling systems (68). Examples are the induction of Epidermal Growth Factor (EGF) signaling in schistosomes by host derived EGF that binds to an evolutionarily conserved EGF receptor kinase (69). We recently demonstrated that also host insulin can stimulate *Echinococcus* development by acting on evolutionarily conserved insulin signaling systems (70). This apparently also extends to cytokines of the FGF family (71) and the TGF-β/BMP family and respective parasite receptors since host BMP2 has been shown to stimulate a TGF-β family receptor kinase of *E. multilocularis* (72) and similar evidence has also been obtained for schistosomes (73). Our own unpublished work further indicates that *Echinococcus* TGF-β receptors can not only interact with host BMP, but also with host TGF-β. It is thus reasonable to assume that parasite-derived cytokines of this family can also functionally interact with TGF-β/BMP receptors of the host. Although we have not yet identified the precise receptor system that is stimulated in T-cells by EmACT, we propose that it acts directly on the Activin receptor-like kinase (Alk) system that is involved in Treg conversion (40, 49, 74, 75). Further investigations as to which mammalian TGF-β/BMP receptor systems are activated by cestode TGF-β family ligands such as EmACT are clearly necessary.

Although the induction of Treg might be beneficial to *Echinococcus* from the very beginning of the infection, we herein mostly focused on E/S products of the metacestode since we previously showed that E/S products of *Echinococcus* primary cells, which functionally resemble the oncosphere-metacestode transition state (6), did not induce Treg conversion (6) and failed to trigger IL-10 release by T-cells (7). The reason for these differences might be different composition of the E/S fractions from metacestode vesicles and early primary cells. Indeed, in transcriptome data collected during the genome project (34), we already observed clear differences between primary cells and metacestodes in the expression of potentially secreted proteins. Furthermore, we also observed that primary cells secrete a factor EmTIP which induces IFN-γ in T-cells and which is not secreted by the metacestode (7). Hence, different stages of the parasite (i.e., less protected (primary cells) and well protected (metacestode) might act differently on T-cells due to a differential E/S profile, and might use different mechanisms for establishing a protective environment. In the case of primary cells, this could include the induction of apoptosis and tolerogenicity in DCs, because they are the first actors at the site of infection (6). In the case of the metacestode, this could, in addition, involve the formation of Tregs in order to not only contain the host response against the actively growing larva, but most probably also to limit extensive tissue damage in the host.

It has been shown that in addition to immunosuppression, chronic AE is also associated with a Th2 immune response (5). This could, in part, result from a dominant Th2 differentiation of Foxp3^+^ Tregs upon loss of Foxp3 expression observed after parasite-driven transient expansion of Foxp3^+^ Tregs after 7 days of infection in our assay. This hypothesis is supported by the reported preferential Th2 differentiation of Treg following Foxp3 loss in human T-cells (76). On the other hand, a certain contribution of EmACT in the Th2 response reported during chronic AE might result from conserved functionalities with mammalian activin A which has been shown to promote, in a context-dependent manner, Th2 effector functions (77, 78).

Taken together, we herein demonstrate that E/S products of the *E. multilocularis* metacestode can actively expand regulatory T-cells of a natural intermediate host and can induce the production of immunosuppressive IL-10 by CD4^+^ T-cells. Among the parasite E/S fraction we identified a structural homolog of mammalian activin A, EmACT, the *in vitro* activities of which indicate that it also functionally resembles TGF-b/activin cytokines of mammals. By releasing EmACT as a supporting E/S factor in inducing Treg and elevated IL-10 production the parasite might actively establishing an immunosuppressive environment during chronic infection.

## Data Availability Statement

The datasets generated for this study can be found in the GenBank HF912278.

## Ethics Statement

The animal study was reviewed and approved by the Ethics Committee of the Government of Lower Franconia, Germany (permit no. 55.2 DMS 2532-2-354).

## Author Contributions

KB, ML, and JN conceived and designed the study, designed the experiments, performed the data analysis, provided reagents, materials, and analysis tools, wrote the manuscript, and read and approved the final manuscript. JN carried out the experiments.

## Conflict of Interest

The authors declare that the research was conducted in the absence of any commercial or financial relationships that could be construed as a potential conflict of interest.

## Footnotes

1 http://www.mybiosoftware.com/alignment/1013

## Supplementary Material

FIGURE S1 Axenization of parasite material. The parasite cysts were harvested from infected jirds and kept under axenic conditions for 10 days. The presence of host contaminants was assessed by organism-specific PCR. Chromosomal DNA was isolated, the host (Jird)-specific ß-tubulin or the parasite-specific elp genes were separately amplified. Jird tissue was used as a negative control for the parasite-specific gene *elp*.

FIGURE S2 Gating Strategy to detect Foxp3 + Regulatory T cells. Lymph node cells cultured for 5 days on CD3/CD28 antibody-coated plates in the presence of TGF-β-containing medium (10 ng/ml) were stained for Foxp3 + regulatory T cell detection by flow cytometry. **(A)** Gating Strategy used to detect Foxp3 + Regulatory T cells. **(B)** Gated CD4^+^ cells analysis by flow cytometry for CD25 and Foxp3 expression. The activated CD25^+^ population is clustered into Teffs (CD25 + Foxp3−) or Tregs (CD25 + Foxp3 +) depending on Foxp3 expression.

FIGURE S3 Transcription of *emact* by *E. multilocularis* larvae. *E. multilocularis* larvae (Primary cells as PC; metacestode vesicles as MV and protoscoleces as Pro) were separately used for qualitative assessment of Em*act* expression. 1μl of each larvae cDNA was used as template for PCR with a high fidelity DNA polymerase (Phusion High-Fidelity DNA Polymerase, New England Biolabs). A 1536 bp product for *Emact* full transcript was amplified using the primers E*mact_Dw* and *Emact_Up* spanning from exons 1–5. 2 μl of each PCR amplicon were resolved on a 1.5% agarose gel and stained with Ethidium bromide prior to visualization under a UV transilluminator.

FIGURE S4 N-term c-myc tagged EmACT secretion pattern in transfected HEK cells. The *Emact*-Psectag2 vector construct was modified by site-directed mutagenesis to incorporate a c-myc tag N-terminal of the EmACT mature peptide sequence and after the furin consensus cleavage motif RTRR. HEK 293 cells were transfected with this construct and kept in culture for collection of supernatant over time (72 h). The collected supernatant was processed using the c-myc tagged protein MILD PURIFICATION KIT Ver.2 (MBL) as per the manufacturer instructions. Briefly, the supernatant was supplemented with anti-c-myc beads for capture of c-myc EmACT mature protein. The incubated beads were eluted with c-myc-containing solutions and the eluate probed with anti-c-myc for myc-tagged proteins.

TABLE S1 List and corresponding accession numbers of gene sequences used.
