# Supplementary figures and images for: Expansion of Host Regulatory T Cells by Secreted Products of the Tapeworm Echinococcus multilocularis
Source: Front Immunol. 2020 May 8;11:798. doi: 10.3389/fimmu.2020.00798 (PMC7225322; doi:10.3389/fimmu.2020.00798)

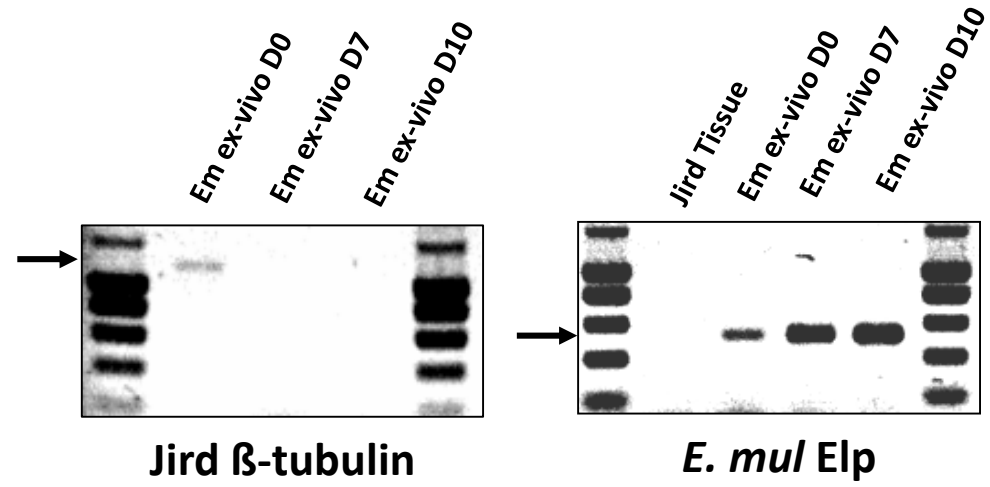

Supplement: FIGURE S1 — Axenization of parasite material. The parasite cysts were harvested from infected jirds and kept under axenic conditions for 10 days. The presence of host contaminants was assessed by organism-specific PCR. Chromosomal DNA was isolated, the host (Jird)-specific ß-tubulin or the parasite-specific elp genes were separately amplified. Jird tissue was used as a negative control for the parasite-specific gene elp. [file Image_1.pdf]

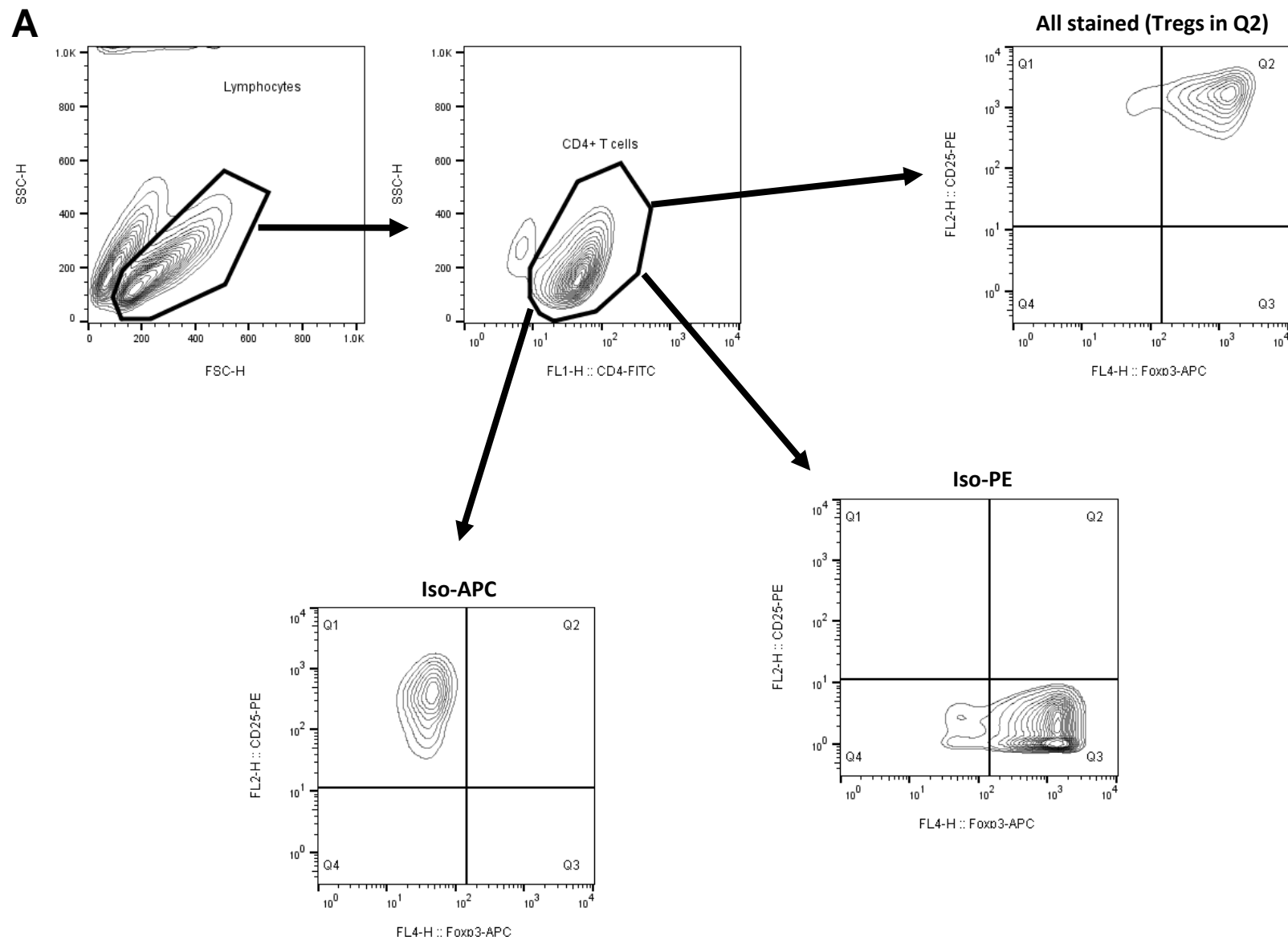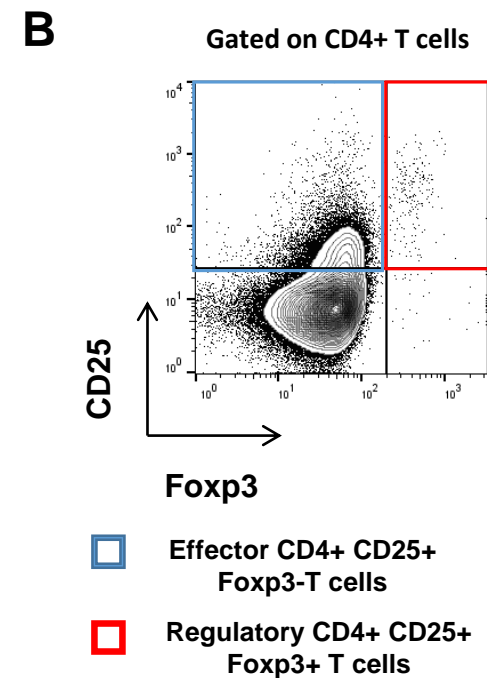

Supplement: FIGURE S2 — Gating Strategy to detect Foxp3 + Regulatory T cells. Lymph node cells cultured for 5 days on CD3/CD28 antibody-coated plates in the presence of TGF-β-containing medium (10 ng/ml) were stained for Foxp3 + regulatory T cell detection by flow cytometry. (A) Gating Strategy used to detect Foxp3 + Regulatory T cells. (B) Gated CD4+ cells analysis by flow cytometry for CD25 and Foxp3 expression. The activated CD25+ population is clustered into Teffs (CD25 + Foxp3−) or Tregs (CD25 + Foxp3 +) depending on Foxp3 expression. [file Image_2.pdf]

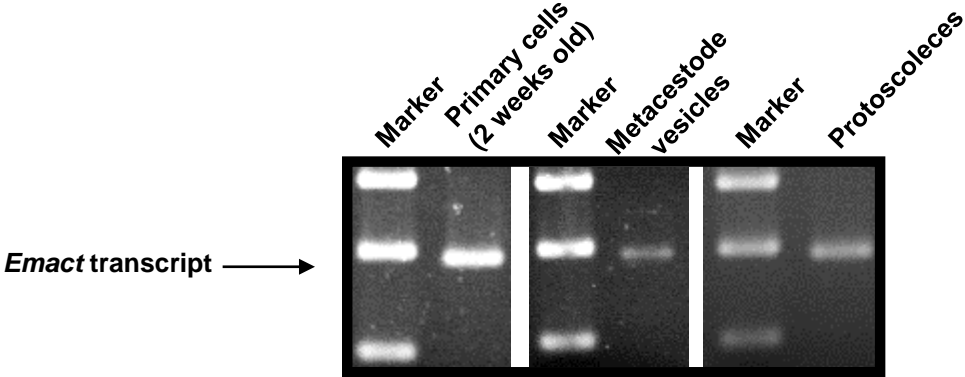

Supplement: FIGURE S3 — Transcription of emact by E. multilocularis larvae. E. multilocularis larvae (Primary cells as PC; metacestode vesicles as MV and protoscoleces as Pro) were separately used for qualitative assessment of Emact expression. 1μl of each larvae cDNA was used as template for PCR with a high fidelity DNA polymerase (Phusion High-Fidelity DNA Polymerase, New England Biolabs). A 1536 bp product for Emact full transcript was amplified using the primers Emact_Dw and Emact_Up spanning from exons 1–5. 2 μl of each PCR amplicon were resolved on a 1.5% agarose gel and stained with Ethidium bromide prior to visualization under a UV transilluminator. [file Image_3.pdf]
