# Supplementary material for: Expansion of Host Regulatory T Cells by Secreted Products of the Tapeworm Echinococcus multilocularis
Source: Front Immunol. 2020 May 8;11:798. doi: 10.3389/fimmu.2020.00798 (PMC7225322; doi:10.3389/fimmu.2020.00798)
Supplement: FIGURE S4 — N-term c-myc tagged EmACT secretion pattern in transfected HEK cells. The Emact-Psectag2 vector construct was modified by site-directed mutagenesis to incorporate a c-myc tag N-terminal of the EmACT mature peptide sequence and after the furin consensus cleavage motif RTRR. HEK 293 cells were transfected with this construct and kept in culture for collection of supernatant over time (72 h). The collected supernatant was processed using the c-myc tagged protein MILD PURIFICATION KIT Ver.2 (MBL) as per the manufacturer instructions. Briefly, the supernatant was supplemented with anti-c-myc beads for capture of c-myc EmACT mature protein. The incubated beads were eluted with c-myc-containing solutions and the eluate probed with anti-c-myc for myc-tagged proteins. [file Image_4.pdf]

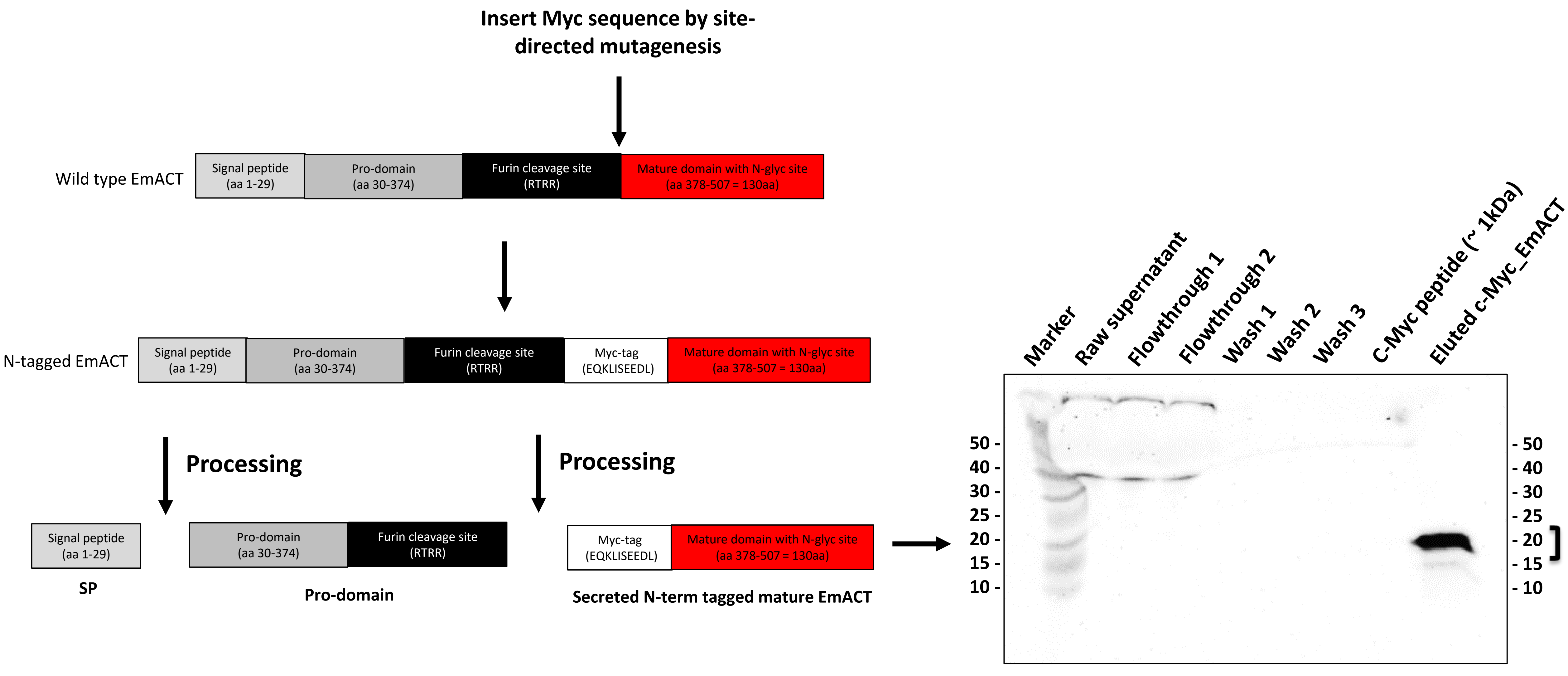

Detection with anti-c-Myc after 60 secondes
